# Impact of Response Stimulus Interval on Transfer of Non-local Dependent Rules in Implicit Learning: An ERP Investigation

**DOI:** 10.3389/fpsyg.2017.02107

**Published:** 2017-12-06

**Authors:** Jianping Huang, Hui Dai, Jing Ye, Chuanlin Zhu, Yingli Li, Dianzhi Liu

**Affiliations:** ^1^Department of Psychology, Soochow University, Suzhou, China; ^2^Department of Psychology, Tsinghua University, Beijing, China; ^3^Student Affairs Office, Nanjing Tech University, Nanjing, China

**Keywords:** implicit sequence learning, transfer, RSI, ERP, non-local dependencies

## Abstract

In the literature on implicit learning, controversy exists regarding whether the knowledge obtained from implicit sequence learning consists of context-bound superficial features or context-free structural rules. To explore the nature of implicit knowledge, event related potentials (ERP) recordings of participants’ performances in a non-local dependent transfer task under two response-stimulus-interval (RSI) conditions (250 and 750 ms) were obtained. In the behavioral data, a transfer effect was found in the 750 ms RSI condition but not in the 250 ms RSI condition, suggesting that a long RSI is the basis for the occurrence of non-local dependent transfer, as which might have provided enough reaction time for participants to process and capture the implicit rule. Moreover, P300 amplitude was found to be sensitive to the impact of RSI on the training process (i.e., the longer RSI elicited higher P300 amplitudes), while variations in both N200 (i.e., a significant increase) and P300 amplitudes (i.e., a significant decrease) were found to be related to the presence of a transfer effect. Our results supported the claim that implicit learning can involve abstract rule knowledge acquisition under an appropriate RSI condition, and that amplitude variation in early ERP components (i.e., N200 and P300) can be useful indexes of non-local dependent learning and transfer effects.

## Introduction

Implicit learning plays an important role in various forms of human cognition, such as language acquisition (Leung and Williams, 2011), music practice (Rohrmeier and Rebuschat, 2012), and the formation of perceptual-motor skills (Fu et al., 2010). As such, implicit learning is a critical component of learning and human life. How does implicit learning occur? According to Cleeremans and Jiménez (2002), representation of implicit knowledge in human brains develops from weak to strong neural association, with its representation quality as well as consciousness improved concurrently. Only knowledge with high quality representation (i.e., in terms of its stability, strength and uniqueness) can be fully conscious and orally reported. Moreover, unconscious knowledge is usually bounded to the stimuli’s perceptual features and thus hard to transfer, while conscious knowledge is often related to embedded rules and thus easy to transfer (Zhang and Liu, 2014).

Several factors may influence knowledge representation quality in implicit learning. Destrebecqz and Cleeremans (2001, 2003) have found that variation in RSI impacts on representation quality of knowledge from implicit learning, with weak representation (i.e., mostly supported by unconscious knowledge) associated with short RSI, and strong representation (i.e., mostly supported by conscious knowledge) associated with long RSI. Similar results have been reported in several other studies (Zhang et al., 2014, 2015).

Another potential factor influencing knowledge representation quality in implicit learning is training length. Despite that Tanaka and Watanabe (2015) found that training length (i.e., number of training trials) failed to predict the occurrence of implicit transfer, other researchers have demonstrated that elongated training length can improve the representation quality of implicit rules and facilitate transfer, with full consciousness (i.e., indexed by accurate oral report) being achieved in the end (Cleeremans and Jiménez, 2002; Destrebecqz and Cleeremans, 2003; Goldstone and Sakamoto, 2003). Notably, in Tanaka and Watanabe’s (2015), study the settings of training length (i.e., 4, 12, 16, and 20 trials, respectively) were probably too short for representation quality to vary among these conditions.

The implicit learning literature suggests that participants are not only able to acquire the perceptual features of implicit rules (e.g., physical properties of stimulus), but also able to obtain the deeper structural knowledge of embedded rules. In the former case, participants acquire local dependencies (i.e., knowledge of perceptual features of stimulus). In the latter case, participants acquire non-local dependencies (i.e., knowledge of abstract rules not dependent on perceptual structures). Early studies on non-local dependent transfer were focused to artificial language learning (Onnis et al., 2005; Williams, 2009), and then expanded to other areas such as implicit music learning and implicit sequence learning. Studies on implicit music learning generally show that tune structures can be implicitly learned and transferred. For example, when studying music tunes, participants were able to detect the change of embedded implicit rules (Dienes and Longuet-Higgins, 2004; Kuhn and Dienes, 2005; Dienes et al., 2012). Li et al. (2013) reported in their study that participants were capable of unconsciously obtaining the structural knowledge of rotated as well as mirror-reversed tunes. Tanaka and Watanabe (2014a,b) demonstrated that participants were able to unconsciously obtain the structural knowledge of spatial and temporal relationship of tunes and transfer it to mirror-reversed or rotated versions. It should be mentioned that these studies used music tunes as the test materials, which are concrete in nature. Therefore, the transfer effect found in those studies may be mediated by concrete knowledge of tunes (e.g., chunks of musical tunes), rather than abstract knowledge of implicit rules. To explore non-local dependent transfer (i.e., transfer of knowledge of implicit rules rather than knowledge of perceptual structures), test materials must be designed to be non-concrete in nature.

The present study was designed to use the technique of ERP to explore the impact of RSI on non-local dependent transfer of implicit sequence learning under extended training (70 trials). Event-related-potentials (ERP) is high in temporal resolution (Abla et al., 2008). It is suitable for exploring brain activation related to tasks associated with quick reactivation, such as implicit sequence learning. Some ERP studies on implicit sequence learning have shown that amplitude variations of the ERP components of N200 and P300 are sensitive to transitions in implicit sequence learning (Miyawaki et al., 2005; Ferdinand et al., 2008; Jost et al., 2011; Fu et al., 2013). There is no previous study using both an implicit transfer paradigm and the technique of ERP to investigate implicit knowledge representation patterns under different RSI conditions. Rüsseler and Rösler (2000) and Rüsseler et al. (2003) have explored knowledge representation pattern differences between implicit and explicit sequence learning by using ERP, and found that implicit sequence learners obtained a response–response connection, while explicit sequence learners obtained one of stimuli–stimuli, stimuli–response, and response–stimuli connections. Moreover, explicit learners’ knowledge was represented as both perceptual and motor patterns, while implicit learners’ knowledge was only represented as motor patterns. These researchers only investigated perceptual and motor implicit rules, but not higher-level abstract implicit rules. Remillard (2008, 2010) explored non-local dependent rule acquisition in a perceptual-motor sequence task and found that participants were capable of acquiring the non-local dependent rule. However, the non-local dependent rule used in Remillard’s study is based on transfer probability and thus may not be suitable for testing abstract rule acquisition in implicit learning. For exploring the possibility of abstract rule acquisition and transfer in implicit learning, a non-local dependent rule with varied first-order structures (i.e., stimulus presentation or perceptual patterns), and an invariant higher-order structure (i.e., an abstract rule) may be a better choice.

Based on the above-mentioned reasons, the present study was aimed to investigate the impact of two different RSIs (i.e., 250 and 750 ms) on non-local dependent transfer in implicit sequence learning by using ERP. The literature suggests that 250 ms RSI and 750 ms RSI are two time settings sensitive to awareness changes, with mostly unconscious knowledge triggered by 250 ms RSI, and a collaboration of conscious and unconscious knowledge triggered by 750 ms RSI (Destrebecqz and Cleeremans, 2001, 2003; Haider and Frensch, 2009; Franco and Destrebecqz, 2012). It was hypothesized in the present study that, only under the condition of 750 ms RSI, participants’ consciousness would continually increase during the training course, and their learning of the implicit rule would gradually develop from perceptual learning to abstract rule learning, which would eventually prompt their behavioral performance from no transfer to transfer. Moreover, Amplitude variations of the two ERP components of N200 and P300 were hypothesized to be sensitive to changes in awareness levels (i.e., consciousness vs. lack of consciousness) and transfer effects.

## Materials and Methods

### Participants

Fifty-five college students (mean age = 21.4, *SD* = 1.43) were randomly selected from a university. All were right-handed and with normal vision. Participants received a small amount of cash as a token of appreciation after they finished the experiment. One participant was excluded from the data because of high error rate on the test (>10%). Another four participants were excluded from the data because of high rates of noise in the ERP data. The final sample consisted of fifty participants (23 males and 27 females). Thirteen participants were randomly assigned to the 250 ms RSI experimental condition, and twelve participants were randomly assigned to the 250 ms RSI control condition. Thirteen participants were randomly assigned to the 750 ms RSI experimental condition, and 12 participants were randomly assigned to the 750 ms RSI control condition (i.e., for the rational of using two control groups, see Materials). All participants voluntarily signed a consent form, which was approved by the Research Ethics Committee of Soochow University. Full ethical review and approval were required according to the national and institutional requirements.

### Materials

This study was conducted in a sound proof room. Participants sat in front of a 17″ computer screen (with a refresh rate of 75 Hz) at a distance of 90 cm. A classic sequence reaction task (Cleeremans and Jiménez, 1998) was adapted for the purpose of the present study. During the task, participants were required to press a key corresponding to the spatial location of a dark dot (with a diameter of 1 cm) presented on a computer screen as quickly and accurately as possible.

**FIGURE 1:**
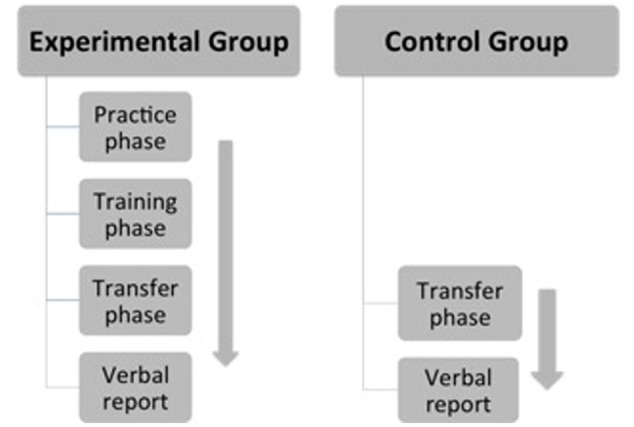
Experimental arrangements for experimental groups and control groups.

The experiment included a training phase and a transfer phase. The two experimental groups were required to finish both the training and the transfer phases, while the two control groups only the transfer phase. The experimental arrangements for both the experimental groups and the control groups were shown in **Figure 1**.

A classic SOC rule (Reed and Johnson, 1994) was used in the sequences, in which the location of a third stimulus is determined by the locations of previous two stimuli (e.g., 34→2, 42→3, 23→1……).

**FIGURE 2:**
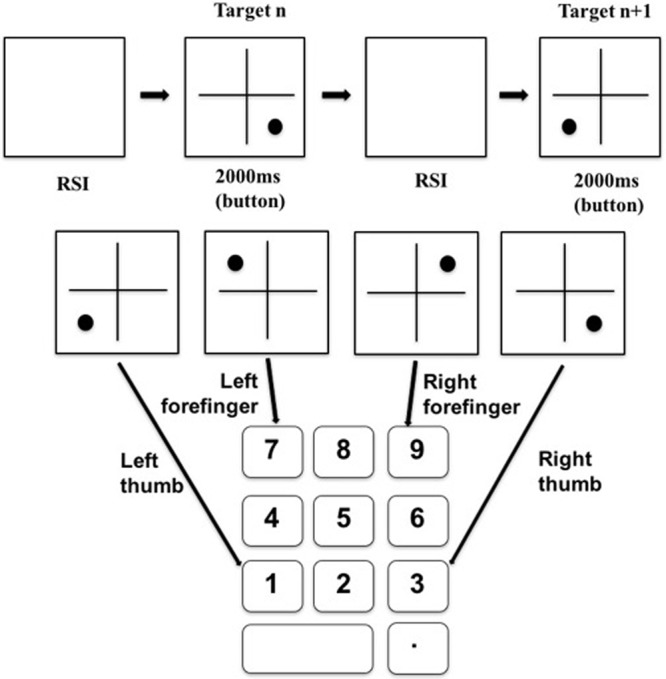
Sample stimulus presentation and corresponding key-press pattern for the training phase.

During the training phase, the spatial location arrangement for the stimuli followed a SOC1 rule: 342312143241 (i.e., numbers represent the four quadrants of the computer screen). Sample presentation sequence and corresponding key-press were shown in **Figure 2**.

**FIGURE 3:**
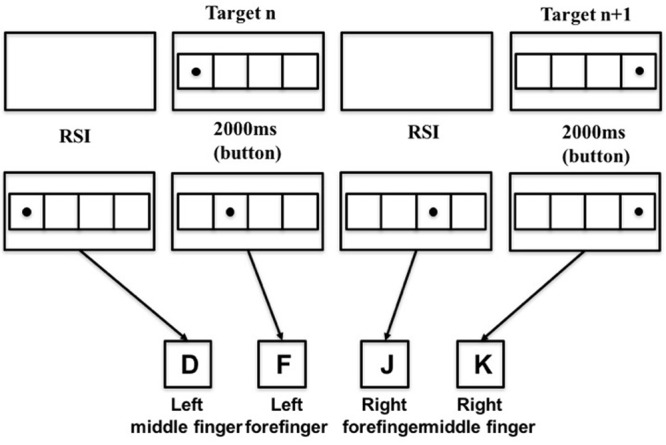
Sample stimulus presentation and corresponding key-press pattern for the transfer phase.

During the transfer phase, the spatial location arrangement for the stimuli followed a SOC2 rule: 341243142132 (i.e., numbers represent the four horizontal locations on the computer screen). Sample presentation sequence and corresponding key-press were shown in **Figure 3**.

Notably, the use of a SOC1 rule for the training phase and a SOC2 rule for the transfer phase are critical for non-local dependent transfer. SOC1 and SOC2 are different in first-order structure (i.e., perceptual features), but share the same higher-order structure (i.e., the SOC rule that the location of a third stimulus is determined by the locations of previous two stimuli). If participants’ knowledge is bounded to perceptual features of SOC1 sequences, transfer to SOC2 sequences is not possible. Successful transfer to SOC2 sequences depends on the acquisition of the higher-order structure of SOC1 sequences. This arrangement is advantageous in comparison to traditional designs, such as using a mirror- reversed rule or a new rule for the transfer phase, in which perceptual transfer and abstract rule transfer can hardly be separated.

Moreover, the setting of control groups in the present study is to control for the possibility that participants are able to acquire the SOC2 rule during the transfer phase, without the training on the SOC1 sequences. If a transfer effect was observed in the experimental groups, with no significant learning effect observed in the control groups (who were tested on the transfer phase only), this transfer effect could be logically inferred as a transfer of abstract knowledge of SOC1 sequences to SOC2 sequences.

All sequences were programmed via E-prime 2.0

### Procedure

All participants received a practice section (24 random trials) before the formal test to be acquainted with the key-pressing.

The training phase consisted of ten blocks, with ninety-six trials in each block. All the blocks (except Block 8) in the training phase were regular blocks. Each regular block contained seven repeated SOC1 sequences and one stochastically inserted random sequence (for the rationale behind inserting a random sequence, see Norman et al., 2007). Block 8 was a random block containing eight random sequences. Participants’ average reaction time in Block 8 was used as a baseline for the training phase. Participants were allowed to rest 15 s between every two blocks. Their accuracy and reaction time were recorded. Logically, a learning effect (i.e. knowledge acquisition by repeated exposure to SOC1 sequences) is indexed by participants’ increasingly shorted reaction time during the training phase. Therefore, implicit learning magnitude was estimated by participants’ average reaction time difference between the random block (i.e., baseline) and its proximate blocks [i.e., *RT*_8_-(*RT*_7_+*RT*_9_)/2].

The transfer phase consisted of six blocks (block 11–block 16), with ninety-six trials in each block. All the blocks (except Block 14) were regular blocks. Each regular block contained seven repeated SOC2 sequences and one stochastically inserted random sequence. Block 14 was a random block containing eight random sequences. Participants’ average reaction time in Block 14 was used as a baseline for the transfer phase. Participants were allowed to rest 15 s between every two blocks. Their accuracy and reaction time were recorded. Logically, a transfer effect (i.e., application of SOC1 knowledge to SOC2 sequences) is indexed by a quick learning (i.e., faster than the control groups, who were not trained on SOC1 sequences) on SOC2 sequences. Therefore, implicit transfer magnitude was estimated by participants’ average reaction time difference between the random block and its proximate blocks [i.e., *RT_14_*-(*RT_13_*+*RT_15_*)/2].

After the test, all participants were asked the following three questions to measure their awareness levels (i.e., consciousness vs. lack of consciousness): what determines the spatial locations of the dots? Can you describe the rule underlying the spatial locations of the dots? By what time during the test did you find out this rule?

### ERP Recording and Analysis

Participants’ EEG data was concurrently recorded during the test by using a 32-channel cap (Brain Product, Munich, Germany). The electrodes on the cap were positioned according to the international 10–20 system. VEOG and HEOG were recorded. The EEG signals were filtered with a bandpass of 0.05–100 Hz and sampled with a rate of 500 Hz.

The original EEG data was processed with the standard procedures provided by the software of ANALYZER 2.0. EEG data corresponding to behavioral data containing more than 10% error responses (Weiermann et al., 2010) and extreme reaction time (i.e., shorter than 100 ms or longer than 1000 ms) was excluded. After eye blink correction, other artifacts (i.e., epochs with EEG power exceeding ± 100 microvolt) were removed from the EEG data. The artifact-free data was segmented into EEG epochs (i.e., 900 ms post-stimulus intervals), baseline corrected (200 ms pre-stimulus interval), and averaged. The ERP data was further divided into six parts corresponding to three learning stages [stage 1 (Block 1, 2, and 3), stage 2 (Block 4, 5, and 6), and stage 3 (Block 7, 9, and 10)] and three transfer stages [stage 4 (Block 11 and 12), stage 5 (Block 13 and 14), and stage 6 (Block 16)]. Following previous studies (Eimer et al., 1996; Schlaghecken et al., 2000), EPR data on the four central electrodes (i.e., Fz, FCz, Cz, Pz) was used in the analysis. The amplitudes of N200 (230–310 ms) and P300 (340–530 ms) were extracted separately for the 250 ms RSI condition and 750 ms RSI condition.

## Results

### Behavioral Data

The oral reports showed that none of the participants were able to accurately describe the implicit rules, indicating that the learning process was implicit for all participants. Behavioral data containing more than 10% error responses and extreme reaction time (i.e., shorter than 100 ms or longer than 1000 ms) was excluded.

#### Occurrence of Implicit Learning on SOC1 Sequences for the Two Experimental Groups

**FIGURE 4:**
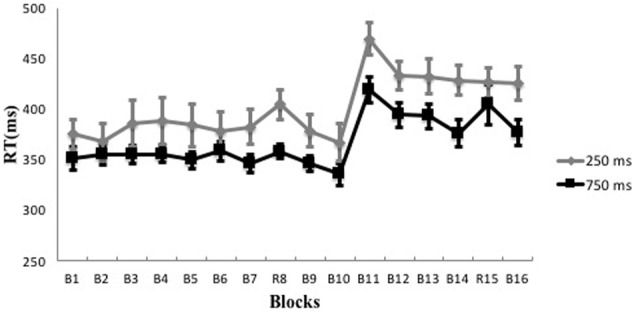
Reaction time trends over all the blocks in the 250 and 750 ms RSI conditions. Error bar indicates the SE.

Participants’ reaction time showed an increase during both the 250 and 750 ms RSI conditions (see **Figure 4**). Based on the commonly used index of implicit learning magnitude [average reaction time difference between the random block and its proximate blocks in the training stage, i.e., *RT*_8_-(*RT*_7_+*RT*_9_)/2 for the present study], average reaction time for Block 8 (*RT*_8_) was compared to average reaction time of its proximate blocks [(*RT*_7_+*RT*_9_)/2]. The occurrence of implicit learning would be indicated by a significantly longer average reaction time of Block 8 than that of its proximate blocks. Repeated ANOVAs with Block as the independent variable showed that the averaged reaction time of Block 8 (404.32 ms ± 54.28 ms) was significantly longer than the average reaction time of its proximate blocks (379.99 ms ± 60.58 ms) for the 250ms RSI experimental group [*F*(1,12) = 22.27, *p* < 0.001, η^2^ = 0.65], and that the averaged reaction time of Block 8 (357.98 ms ± 23.56ms) was significantly longer than the average reaction time of its proximate blocks (345.94 ms ± 29.87 ms) for the 750 ms RSI experimental group [*F*(1,12) = 10.73, *p* < 0.01, η^2^ = 0.47]. These results suggest that effective implicit learning occurred in both experimental groups.

#### Comparison of Implicit Learning Magnitudes between the Two Experimental Groups

To further explore the relative amount of implicit learning magnitudes between the two experimental groups, a one-way ANOVA with Group as the independent variable and learning magnitude as the dependent variable showed that the 250 ms RSI and 750ms RSI experimental groups (24.33 ms ± 18.59 ms vs. 12.04 ms ± 13.29 ms) did not differ significantly on learning magnitude [*F*(1,12) = 2.38, *p* > 0.05, η^2^ = 0.17].

#### Comparison of Implicit Transfer Magnitudes between the Two Experimental Groups

To explore transfer effects in the two experimental groups, repeated ANOVAs with Block as the independent variable were conducted to compare the mean reaction time of Block 14 (*RT_14_*) to the mean reaction time of its proximate blocks [*(RT_13_ + RT_15_)/2*]. These analyses yielded a significant main effect for the 750 ms RSI experimental group [*F*(1,12) = 9.09, *p* < 0.05, η^2^ = 0.43], showing longer reaction time in Block 14 (404.32 ms ± 70.99 ms) than that in its proximate blocks (376.17 ms ± 46.06 ms). No significant difference was found for the 250 ms RSI experimental group [*F*(1,12) = 0.01, *p* > 0.05, η^2^ = 0.001]. These results suggest that effective transfer occurred in the 750 ms RSI experimental group, but not in the 250 ms RSI experimental group.

#### Implicit Learning Magnitudes in the Control Groups

In the above analysis, the observed significant reaction time changes in the 750 ms RSI experimental group during the transfer phase can be caused by both a transfer of SOC1 knowledge to SOC2 sequences (i.e., a transfer effect) and the learning process on SOC2 sequences (i.e., a learning effect). To examine whether it is a transfer effect or a learning effect, data of the control groups must be compared to that of the experimental groups. Notably, participants in the 250 and 750 ms RSI control groups were assigned to the transfer phase without any previous training. If the control groups fail to show a significant learning effect on SOC2 sequences, it can be inferred that the observed significant reaction time changes in the 750 ms RSI experimental group is a transfer effect rather than a learning effect.

Repeated ANOVAs for the control groups revealed no significant learning effects in both the 250ms RSI control group (493.81 ms ± 79.48 ms vs. 481.94 ms ± 76.02 ms) [*F*(1,11) = 2.54, *p* > 0.05, η^2^ = 0.19] and the 750 ms RSI control group (425.44 ms ± 44.17 ms vs. 423.09 ms ± 47.42 ms) [*F*(1, 11) = 0.13, *p* > 0.05, η^2^ = 0.01]. These results suggested that 4 blocks of SOC2 sequence training were not enough for the control groups to capture the structural rule, and confirmed that the reaction time decrease over the transfer phase in the 750 ms RSI experimental group was a transfer effect (i.e., the transfer of their previous SOC1 knowledge to SOC2 sequence learning), rather than a learning effect (i.e. structural rule acquisition by exposure to the 4 blocks of SOC2 sequences).

### ERP Data

#### ERP Data for the Training Phase

For the experimental groups, a 2 (Condition: 250 ms vs. 750 ms) × 4 (Electrode: Fz, Cz, Pz, and FCz) × 3 (Stage: 1, 2, and 3) mixed ANOVA with N200 amplitude as the dependent variable was conducted. This analysis showed a significant main effect for Electrode [*F*(3,216) = 12.21, *p* < 0.001, η^2^ = 0.15], with N200 amplitude over Fz being significantly stronger than those over the other three electrodes (*p*s < 0.001). No other significant effect was found. Notably, the lack of a significant main effect of Condition suggests that N200 component is not sensitive for differentiating the implicit learning processes between the 250 and 750 ms RSI conditions.

**FIGURE 5:**
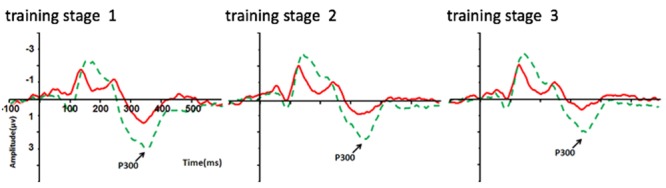
P300 amplitude (μV) differences on the electrode of Pz between the 250 and 750 ms RSI groups over the training phase (three stages). Red line = 250 ms RSI group; Green line = 750 ms RSI group.

**FIGURE 6:**
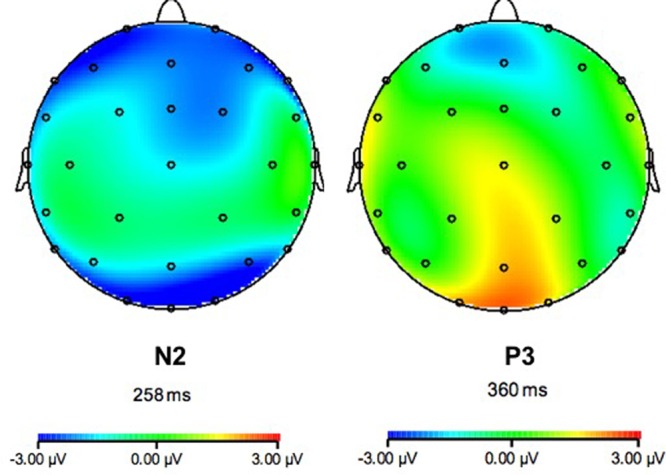
Scalp distribution of N200 and P300 in the 750 ms RSI group over the transfer phase.

The same mixed ANOVA with P300 amplitude as the dependent variable was performed for the experimental groups. This analysis showed a significant main effect for Electrode [*F*(3,216) = 12.09, *p* < 0.001, η^2^ = 0.14], with P300 amplitude over Fz being significantly weaker than those over the other three electrodes (*p*s < 0.001; Bonferroni corrected). A significant main effect for Condition was also found [*F*(1,72) = 6.38, *p* < 0.05, η^2^ = 0.08], with stronger P300 amplitude shown in the 750 ms RSI condition (0.86 μV ± 1.41 μV) than that in the 250 ms RSI condition (0.10 μV ± 0.88 μV) (see **Figures 5, 6**), suggesting that P300 amplitude was sensitive to different learning processes between the two RSI conditions. No other significant effect was found.

#### ERP Data for the Transfer Phase

To explore the sensitivity of N200 and P300 in detecting transfer effect, experimental groups and control groups were compared on their ERP signals in the transfer phase.

##### N200 amplitude differences over the transfer phase

For the 250 ms RSI condition, a 2 (Group: experimental group vs. control group) × 4 (Electrode: Fz, Cz, Pz, FCz) × 3 (Stage: 4, 5, and 6) mixed ANOVA with N200 amplitude as the dependent variable was conducted. This analysis showed a significant main effect for Electrode: *F*(3,276) = 13.16, *p* <0.001, η^2^ = 0.16, with N200 amplitude over Fz (-2.06 μV ± 0.29 μV) being significantly stronger than those over the other three electrodes (-0.62 μV ± 0.31 μV; -0.22 μV ± 0.24 μV; -0.88 μV ± 0.19 μV) (*p*s < 0.001, Bonferroni corrected). A significant Group × Electrode interaction effect was also found: *F*(3,276) = 9.13, *p* < 0.001, η^2^ = 0.12. Follow-up independent *t* tests for this interaction effect showed that N200 amplitude of the experimental group (-3.22 μV ± 3.07 μV) was significantly stronger than that of the control group (-0.89 μV ± 1.59 μV) over Fz [*t*(73) = -4.06, *p* < 0.001, Bonferroni corrected].

For the 750 ms RSI condition, the same mixed ANOVA with N200 amplitude as the dependent variable was conducted. This analysis showed a significant main effect for Electrode [*F*(3,276) = 27.38, *p* < 0.001, η^2^ = 0.28], with N200 amplitude over Pz (0.24 μV ± 2.45 μV) being significantly weaker than that over the other three electrodes (-1.04 μV ± 2.04 μV; -0.85 μV ± 2.23 μV; -1.11 μV ± 2.26 μV) (*p* < 0.001, Bonferroni corrected). A significant main effect for Group was found [*F*(1,69) = 44.38, *p* < 0.001, η^2^ = 0.39], with N200 amplitude of the experimental group being stronger than that of the control group. A significant Group × Electrode interaction effect was also found: *F*(3,276) = 10.6, *p* < 0.001, η^2^ = 0.13. Follow-up independent *t*-tests for this interaction effect revealed that N200 amplitudes of the experimental group (-1.86 μV ± 1.50 μV; -1.49 μV ± 1.31 μV; -2.35 μV ± 1.29 μV) were significantly stronger than those of the control group (0.23 μV ± 2.39 μV; 2.13 μV ± 1.97 μV; 0.23 μV ± 2.32 μV) over Cz, Pz, and FCz [*t*(73) = -4.57, *t*(73) = -9.46, *t*(73) = -6.02, *p*s < 0.01; Bonferroni corrected].

##### P300 amplitude differences over the transfer phase

For the 250 ms RSI condition, 2 (Group: experimental group vs. control group) × 4 (Electrode: Fz, Cz, Pz, FCz) × 3 (Stage: 4, 5, and 6) mixed ANOVA with P300 amplitude as the dependent variable yielded a significant main effect for Electrode [*F*(3,276) = 14.57, *p* < 0.001, η^2^ = 0.17], with P300 amplitudes over FCz (0.43 μV ± 2.13 μV) and Pz (0.01 μV ± 1.62 μV) being significantly stronger that those over Fz (-1.50 μV ± 2.29 μV) and Cz (-0.58 μV ± 1.79 μV) (*p*s < 0.001, Bonferroni corrected). Another significant Group × Electrode interaction effect was also found: *F*(3,276) = 9.65, *p* < 0.001, η^2^ = 0.12. Follow-up independent *t*-tests for this interaction effect showed that P300 amplitude in the control group (-0.56 μV ± 1.44 μV) was stronger than that in the experimental group (-2.38 μV ± 2.59 μV) over Fz [*t*(73) = -3.73, *p* < 0.01, Bonferroni corrected].

For the 750 ms RSI condition, the same mixed ANOVA mixed ANOVA with P300 amplitude as the dependent variable yielded a significant main effect for Electrode [*F*(3,276) = 22.56, *p* < 0.001, η^2^ = 0.25], with P300 amplitude over Pz (0.47 μV ± 1.28 μV) being significantly stronger than those over the other electrodes (-0.54 μV ± 1.37 μV; -0.34 μV ± 1.12 μV; -0.57 μV ± 1.35 μV; *p*s < 0.001). A significant main effect for Group was also found [*F*(1,69) = 14.24, *p* < 0.001, η^2^ = 0.17], which was due to stronger P300 amplitude in the control group than the experimental group. Another significant Group × Electrode interaction effect was also found: *F*(3,276) = 8.81, *p* < 0.001, η^2^ = 0.11. Follow-up independent *t* tests for this interaction effect showed that P300 amplitudes over Fz, Pz, and FCz (-0.26 μV ± 1.53 μV; 1.38 μV ± 0.92 μV; -0.26 μV ± 1.66 μV) were significantly stronger in the control group than those in the experimental group (-0.81 μV ± 1.17 μV; -0.36 μV ± 0.97 μV; -0.86 μV ± 0.92 μV) [*t*(73) = 2.00; *t*(73) = 7.41; *t*(73) = 2.05, *p*s < 0.05; Bonferroni corrected].

## Discussion

### Impact of RSI on Non-local Dependent Transfer

Conflicts regarding the transferability of implicit knowledge can be found in early artificial grammar learning studies. One of the main questions is whether implicit learning involves the acquisition of perceptual patterns or abstract rules. Reber (1967) argued that knowledge obtained from implicit learning was the representation of an abstract rule. However, some other researchers believed that people could only acquire perceptual patterns in implicit learning, such as repeated letter chunks (Perruchet and Vinter, 1998), or specific artificial grammar examples (Vokey and Brooks, 1992; Jamieson and Mewhort, 2009). Both points of view have been supported by some empirical studies (Kuhn and Dienes, 2005, 2006, 2008; Pothos, 2007). However, neither of them has been fully backed up. In light of this, we used a completely abstract non-local dependent rule in the present study. In this non-local dependent design, the SOC2 rule in the transfer phase shares a higher-order structural rule (i.e., the location of a third stimulus is determined by the locations of previous two stimuli), rather than superficial representations with the SOC1 rule in the training phase. Therefore, a transfer effect depends on the acquisition of the higher-order structure, rather than superficial patterns of the training sequences. Our behavioral results showed a significant transfer effect in the 750 ms RSI condition, with no accurate description of the implicit rule being reported, indicating that participants were able to acquire the deep structural rule embedded in repeated sequences without full consciousness. The fact suggests that implicit knowledge can be abstract and transferable in nature under an appropriate RSI condition.

Notably, no significant non-local dependent transfer was found in the 250 ms RSI condition. Previous studies suggest that 250 ms RSI elicits primarily unconscious knowledge, which is bounded to perceptual patterns of sequences and therefore hard to transfer (Cleeremans and Jiménez, 2002; Kuhn and Dienes, 2006; Zhang and Liu, 2014). With elongated RSI, the implicit learning process tends to involve more and more conscious knowledge (i.e., abstract and transferable knowledge), and eventually leads to an effective transfer (Kuhn and Dienes, 2006). Consistently, the absence of non-local dependent transfer in the 250 ms RSI condition in the present study may be related to overly low representation quality of implicit knowledge caused by a limited RSI.

Interestingly, in the present study, no significant learning effects were found in the control groups with 3 blocks of training on SOC2 sequences. This result stands contrast to the significant learning effects with 7 blocks of training on SOC1 sequences in the experimental groups (i.e., with relatively similar sample sizes in the experimental groups and control groups). This finding is consistent with the reports in previous studies, as such that effective implicit learning could only occur with appropriate length of training (Cleeremans and Jiménez, 2002; Destrebecqz and Cleeremans, 2003; Goldstone and Sakamoto, 2003; Cleeremans, 2006). Our finding suggests that a qualitative difference of non-local dependent learning effect can occur between 3 blocks (i.e., 288 trials) and 7 blocks of training (672 trials). However, since no detection of learning effect in the early training stage was set in the present study experimental design, it could not tell us the exact numbers of training trial under which the transition occurred. This query may be explored in future studies using a revised design to detect the occurrence of effective learning in early stage of non-local dependent training.

### Sensitivity of P300 to Changes in the Training Phase

N200 has been though as an index of early processing (i.e., coding and storing) of sequence information (Fu et al., 2013). In the present study, N200 amplitude during the training phase was not significantly different between the 250 and 750 ms RSI experimental groups, suggesting that it is not sensitive for detecting RSI variation. However, this result may also suggest that RSI variation does not affect early processing of sequence information.

P300 has been suggested as indicating participants’ subjective estimation of the material being processed, which is closely related to increased consciousness (Stadler et al., 2006). Similar to previous reports (Rugg et al., 1998; Atienza et al., 2003; Chu and Liu, 2010), P300 amplitude was found to be significantly stronger in the 750 ms RSI experimental group than that in the 250 ms RSI experimental group in the present study, which may be caused by the increase of consciousness in the 750 ms RSI experimental group (i.e., despite that no full consciousness was achieve in this group). Moreover, given that effective transfer effect was only found in the 750 ms RSI experimental group, its strengthened P300 amplitude relative to that in the 250 ms RSI experimental group during the training phase may suggest a learning process involves abstract rules rather than perceptual patterns.

### Dissociated Transfer Effects Indexed by ERP Components

Our ERP data of the transfer phase showed that N200 amplitude in the experimental group was significantly stronger than that in the control group over Fz in the 250 ms RSI condition, and over all central electrodes in the 750 ms RSI condition According to Mecklinger (2010), N200 is related to participants’ prediction in sequence learning. Strengthened N200 amplitudes in the experimental groups may indicate that the experimental groups were more prone to making prediction of stimulus’ location in the SOC2 sequences based on their knowledge of the SOC1 sequences, while the control groups tended to perceive the SOC2 stimuli as randomly presented.

In contrast, P300 amplitudes in the experimental groups were significantly weaker than those in the control groups during the transfer phase in both 250 and 750 ms conditions. According to Kok (2001), increase of P300 amplitude over the central electrodes indicates an increase of attention resources. Therefore, this result may suggest that the control groups activated more attention resources than the experimental groups to process the sequences, which was completely novel to them.

## Conclusion

Overall, the present study preliminarily proved that a non-local dependent design was useful for differentiating the confounding effects of perceptual similarity and structural identical in traditional study paradigms.

Our behavioral results showed that, with enough processing time (i.e., a 750 ms RSI), participants were capable of acquiring abstract and transferable implicit knowledge of non-local dependencies. Our ERP data showed that both N200 and P300 were useful for detecting non-local dependent transfer effect. Increase in N200 amplitude indicates enhanced ability to predict stimuli sequences and decrease in P300 amplitude indicates less attention and effort needed. Moreover, Increase in P300 amplitude may suggest that the learning process involves the acquisition of abstract knowledge (i.e., implicit rules) rather than perceptual knowledge. Moreover, Both N200 amplitude and P300 amplitude varied significantly between the experimental and the control groups, suggesting that they are useful in differentiating a transfer process and a learning process.

Despite the fact that none of the participants achieved full consciousness (i.e., based on their oral reports), training on SOC1 sequences was found to facilitate study on SOC2 sequences under the 750 ms RSI condition, suggesting that abstract knowledge can be acquired with partial consciousness. However, it is unknown whether SOC1 training could continually facilitate the study on SOC2 sequences, or only be temporally effective in the early stage. This question can be explored in follow-up studies using more SOC2 blocks.

## Bullet Points

(1) Longer RSI increases the chance of successful non-local dependent transfer.
(2) Increase in P300 amplitude during the training phase indicates a learning of abstract rules rather than perceptual knowledge.
(3) Increase in N200 amplitude and decrease in P300 amplitude during the transfer phase indicate a transfer effect of SOC1 training to SOC2 learning.

## Author Contributions

Guarantor of integrity of entire study: DL. Study concepts: JH. Study design: JH and HD. Literature research: JH. Clinical studies: JH. Experimental studies: JH and HD. Data acquisition: JH, HD, and JY. Data analysis/interpretation: JH and CZ. Statistical analysis: JH. Manuscript preparation: JH and CZ. Manuscript definition of intellectual content: JH, YL, and DL. Manuscript editing: JH, YL, and DL. Manuscript revision/review: YL and DL. Manuscript final version approval: JH, YL, and DL.

## Acknowledgment

This research was supported by National Natural Science Foundation of China under Grant 31271084 and Grant 31540025.

## Conflict of Interest Statement

The authors declare that the research was conducted in the absence of any commercial or financial relationships that could be construed as a potential conflict of interest.
